# Combined Effects of Different Bracket Bonding Adhesives and Different Resin Removal Methods on Enamel Discoloration: A Preliminary Study

**DOI:** 10.1155/2023/8838264

**Published:** 2023-02-16

**Authors:** Ozra Niknam, Milad Shamohammadi, Zeynab Ataei, Vahid Rakhshan

**Affiliations:** ^**1**^ Department of Orthodontic, School of Dentistry, Ahvaz Jundishapur University of Medical Sciences, Ahvaz, Iran; ^2^School of Dentistry, Ahvaz Jundishapur University of Medical Sciences, Ahvaz, Iran; ^3^Department of Anatomy, Dental School, Azad University of Medical Sciences, Tehran, Iran

## Abstract

**Introduction:**

This study aimed to investigate the effects of 3 orthodontic bracket adhesives and 3 resin removal methods on enamel discoloration.

**Methods:**

Ninety metal orthodontic brackets were bonded to 90 intact human premolars, using 3 adhesives (total etch composite (Transbond), self-etch composite (OptiBond), and light-curedresin-modified glass ionomer cement (RMGI, Fuji); *n* = 3 × 30). Each “bracket bonding” group (*n* = 30) was randomly divided into three subgroups of 10 specimens each, each with a different method of remnant resin removal (using only tungsten carbide burs; using tungsten carbide burs plus Sof-Lex polisher discs; using tungsten carbide burs and Stainbuster burs; *n* = 3 × 30). After bracket debonding and coffee staining (at 37°C for one week), the color change parameters (Δa, Δb, ΔL, and ΔE) were measured and then analyzed statistically (*α* = 0.05).

**Results:**

All 9 mean ΔE values were significantly greater than 3.7 and 1.0 (*P* values ≤0.002, *t*-test). The effects of composites and resin removal methods on the ΔE parameter (and their interaction) were significant (*P* values ≤0.008, two-way ANOVA). There were significant pairwise comparisons between total etch (Transbond) and each of the other composites (*P* values ≤0.008, Tukey). Nonetheless, the difference between self-etch (OptiBond) and RMGI (Fuji) was not significant (*P*=0.967). There were significant pairwise comparisons between the ΔE parameter of group “Bur + Stainbuster” and ΔE of each of the other methods (both *P* values ≤0.017).

**Conclusions:**

All 9 pairs of adhesives and resin removal techniques will cause quite visible discolorations. Still, self-etch composites or RMGI might be recommended over total etch composites. Moreover, using Stainbuster burs together with tungsten carbide burs is recommended to reduce discoloration. However, the coloration caused by each composite type can change drastically given the following adhesive removal technique used.

## 1. Introduction

Orthodontic brackets and their removal may cause damage to the enamel as a direct result of dental enamel manipulation or conditions altered by bonding agents [1]. Bonding and debonding processes may wear off about 10–20% of the enamel surface and cause changes in its appearance [2].

The connection between orthodontic resins and the enamel needs to be temporary but at the same time strong enough to withhold orthodontic forces [3–6]. Therefore, manufacturers try to improve the properties of orthodontic adhesives [5]. Bracket bonding can be improved by various factors such as optimizing the additional materials within the adhesive, using proper adhesive types, performing appropriate surface treatments, avoiding contaminants (e.g., blood or saliva), or applying antioxidants [3–10].

Moreover, the removal of adhesive remnants can change the color of the enamel because the resin irreversibly penetrates the enamel and changes its internal color, not to mention the formation of white spot [11, 12]. The search for an efficient and safe method to remove remnant adhesives has led to the introduction of various techniques for resin removal, which include scraping the surface with a scaler to remove resin, removal with tungsten carbide burs or diamond burs, using polishing discs and paste, air abrasion techniques, and ultrasonic application [13–15]. Despite all the studies in this regard, a safe and effective technique for resin removal has not been determined yet. Many dentists try to remove residual resin without sufficient knowledge and based on trial and error, which in some cases can damage the teeth [16]. Even under laboratory conditions, it is virtually impossible to remove the entire adhesive residue from the enamel surface without the aid of high magnification [17].

Therefore, the availability of an orthodontic bonding agent with minimal discoloration and the ability to remove its residue by following a simple protocol would be highly desirable. The literature on the effect of orthodontic bracket bonding/removal on enamel discoloration is scarce and controversial [18, 19]. Moreover, no study has simultaneously examined both factors affecting the colorability of teeth after bracket debonding (bonding factors and resin removal factors): all of the few previous studies had either investigated bonding factors or resin removal factors but not both. Besides, no studies have investigated dental discoloration with Bis-GMA composites. Therefore, this study was conducted to simultaneously assess all the factors that could play a role in the colorability of teeth (both in the bonding and debonding stages). The null hypotheses were the lack of any significant difference between the groups of different adhesives and different resin removal methods or different pairs of “adhesives and resin removal methods” in terms of any of the colorimetry parameters.

## 2. Materials and Methods

This experimental *in vitro* study was performed on 90 intact human premolars. The teeth had been extracted solely for orthodontic treatment purposes. Therefore, no harm was done to any individuals. The ethics of the study were approved by the research committee of the university (ethics code: IR.AJUMS.REC.1399.453). The inclusion criteria comprised intact buccal enamels free of caries and without any hypocalcification or fluorosis on the buccal enamel surface. The extracted premolars were preserved in a 10% formalin solution until all the teeth were collected.

Similar to all previous studies, there was no mathematical sample size calculation. Instead, we used a rule of thumb and improved it: the sample size for dental colorimetry studies is usually determined as 3 to 6 specimens per group. In this study, this routine number was increased to 10 specimens per group in order to improve the reliability of the findings. There were 9 groups, which accounted for 90 specimens.

### 2.1. Bracket Bonding Groups

All the experiments were performed according to the manufacturers' instructions. All the brackets used in all 90 specimens were MBT, 0.022” metal brackets (Fairfield Orthodontics, Fairfield West Plaza, CA, USA). First, the teeth were randomly divided into 3 “bracket bonding” groups of 30 specimens each. In each group, a different adhesive material was used for bonding the metal orthodontic brackets.

#### 2.1.1. Group 1 (Total Etch Composite)

In this group, enamel etching was performed using 37% phosphoric acid for 30 seconds. Then, the teeth were washed for 10 seconds and sprayed with air for 10 seconds. Bonding of the bracket to the tooth was performed using Transbond MIP Primer adhesive material (3M UNITEK, Monrovia, California, USA) containing primer: TEGDEMA and Bis-GMA and Transbond XT resin composite (3M UNITEK) containing Bis-GMA and TEGDEMA. This was performed by applying an adhesive layer on the buccal surface of the tooth and light-curing it for 6 seconds (from a 1 mm distance) using an LED light-curing device (LED.H Ortho, Woodpecker, Beijing, China). Afterward, the composite was placed over the cured adhesive, and the bracket was placed on it. After placing the bracket, the composite resin was light-cured for 20 seconds (10 seconds from the mesial side and 10 seconds from the distal side).

#### 2.1.2. Group 2 (Self-Etch Composite)

In this group, a 7th-generation bonding agent (self-etching primer, OptiBond All in One, Kerr, CA, USA) was used and no separate etching was done. First, the tooth surface was washed and dried (as mentioned in Group 1). Then, the self-etch adhesive was scrubbed on the buccal surface of the tooth for 20 seconds. It was then gently air-sprayed for 5 seconds. Then, it was light-cured for 10 seconds (5 seconds from the mesial side and 5 seconds from the distal side). Next, Transbond XT composite resin (3M Unitek) was used to bond the brackets; after placing the bracket over the smeared composite, it was cured for 20 seconds (as mentioned previously).

#### 2.1.3. Group 3 (Light-Activated Resin-Modified Glass Ionomer Cement)

First, 10% polyacrylic acid was applied for 20 seconds on the tooth surface. Then, the teeth were washed for 15 seconds, and the tooth surface was kept moist. Then, light-activated resin-modified glass ionomer cement (RMGI, Fuji ORTHO LC, GC, Tokyo, Japan) was applied to the enamel as an adhesive material for bonding the brackets. For this purpose, the RMGI adhesive was applied to the buccal surface; then, the bracket was placed on it, and the adhesive was light-cured for 40 seconds (20 seconds from the mesial side and 20 seconds from the distal side).

### 2.2. Bracket Debonding

After the bonding process, the teeth were kept in artificial saliva for 48 hours. After that, the brackets were debonded by special orthodontic pliers for bracket removal (straight, Premium-Line, Dentaurum, Ispringen, Germany).

### 2.3. Resin Removal Groups

In the next step, each of the abovementioned “bracket bonding” groups (*n* = 30) was randomly divided into three subgroups of 10 specimens each, each with a different method of remnant resin removal (or clean-up process). In this way, the whole sample of 90 specimens was divided into 3 “resin removal” groups of 30 specimens each. The 3 methods of resin removal were

#### 2.3.1. A

Tungsten carbide burs (debonding carbide bur, coarse, Dentaurum) attached to a high-speed handpiece at 40000 rpm, without water irrigation.

#### 2.3.2. B

Tungsten carbide bur (debonding carbide bur, coarse, Dentaurum) attached to a high-speed handpiece at 40000 rpm, without water spraying, and then polishing the surface with Sof-Lex polisher discs (3M).

#### 2.3.3. C

Tungsten carbide burs (debonding carbide bur, coarse, Dentaurum) attached to a high-speed handpiece at 40000 rpm, without water flow, and then polishing the enamel using silica burs (#2505, Stainbuster, Dentatus, Hawthorne, NY, USA).

### 2.4. Colorimetry

#### 2.4.1. Initial Colorimetry

In order to determine the initial color using the colorimetry parameters (L, a, and b), after resin removal, the teeth were numbered and then photographed with a digital camera (Nikon, Tokyo, Japan), and the images were transferred to the computer. The images were processed with the help of Photoshop 2018 software (Adobe, San Jose, California, USA), and their primary color was determined. The “a” parameter is a chromatic coordinate for the green-red spectrum, defined as a spectrum of negative values (green) to positive values (red). The “b” parameter is a chromatic coordinate for the blue-yellow spectrum, defined as a spectrum of negative values (blue) to positive values (yellow). The “L” parameter is an indicator of brightness with 0–100 meaning absolutely dark to absolutely bright, respectively.

#### 2.4.2. Caffeine Staining

Then, the teeth were stored in a caffeine solution for one week. The teeth were stored in 100 ml of caffeine solution (Nescafe Classic, Nestle, Switzerland) at 37°C for one week in 9 separate containers (each container for one subgroup of 10). Every day, the caffeine solution was replaced with a new one.

#### 2.4.3. Secondary Colorimetry

After one week in the caffeine solution, the teeth were cleaned with deionized distilled water. Then, to evaluate the colorimetry parameters (L, a, and b), a digital photo was taken with a Nikon camera. The image was evaluated in Photoshop software. To standardize the photography conditions, the teeth were placed in the same position with an indirect light source on a black background and at an angle of 90 degrees to the camera.

#### 2.4.4. Differential Colorimetry

Finally, differential colorimetry was performed by comparing the data obtained from the L, a, and b parameters for the images before and after exposure to caffeine. The extent of the color difference was calculated using the following formula:

(1) $$\begin{array}{c} ∆E=\sqrt{\left[{\left(∆L^{*}\right)}^{2}+{\left(∆a^{*}\right)}^{2}+{\left(∆b^{*}\right)}^{2}\right].} \end{array}$$

ΔE values less than 1 are regarded as color matches; there is a consensus that only ΔE values above 3.7 are considered clinically observable differences [12, 20].

### 2.5. Statistical Analyses

Descriptive statistics and 95% confidence intervals (CIs) were calculated for various parameters. Data normality was examined and passed using a Kolmogorov–Smirnov test and histogram inspection. A two-way analysis of variance (ANOVA) and a Tukey post hoc test were used to compare the groups with each other in terms of their Δa, Δb, ΔL, and ΔE values. A one-sample *t*-test was used to compare ΔE values with values 1.0 and 3.7. Since the interactions had become significant, subgroup analyses were conducted. As subgroup analyses, a one-way ANOVA followed by a Tukey test was used to compare baseline values as well as Δa, Δb, ΔL, and ΔE values of different resin removal methods within each composite. Finally, the one-way ANOVA and Tukey test were used to compare ΔE values within the 9 groups. The software package in use was SPSS 25 (IBM, Armonk, NY, USA). The level of significance was set at 0.05.

## 3. Results

**Figure 1:**
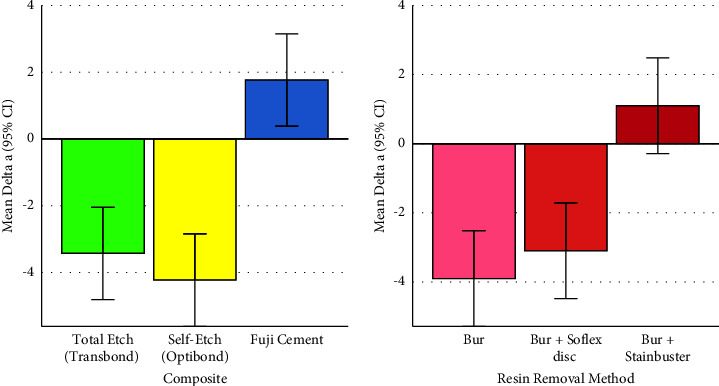
Means (and 95% CIs) for the Δa parameter in 3 composites (*n* = 3 × 30) and 3 adhesive removal methods (*n* = 3 × 30). Positive values indicate shades of red, while negative values indicate shades of green.

**Figure 2:**
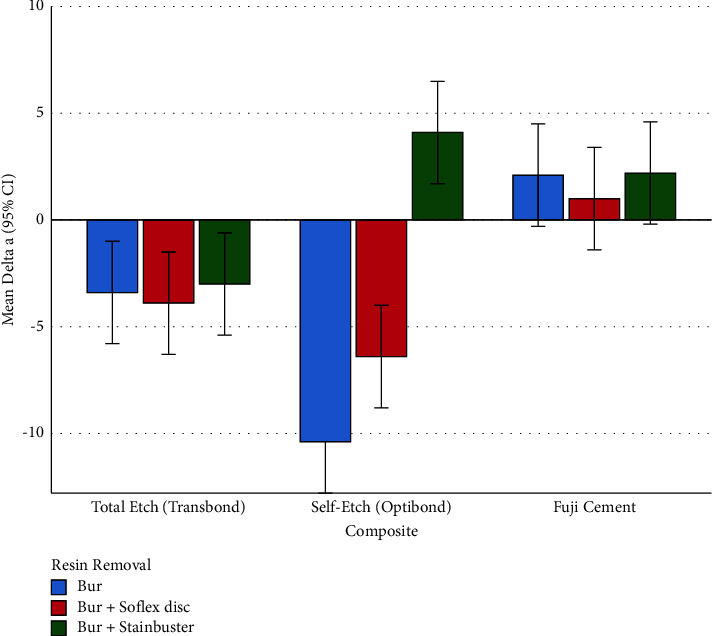
Means (and 95% CIs) for the Δa parameter in all the 9 groups (*n* = 9 × 10). Positive values indicate shades of red, while negative values indicate shades of green.

**Figure 3:**
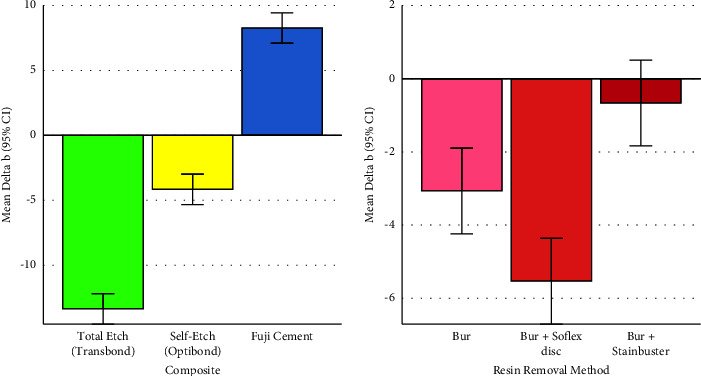
Means (and 95% CIs) for the Δb parameter in 3 composites (*n* = 3 × 30) and 3 adhesive removal methods (*n* = 3 × 30). Negative values indicate shades of blue, while positive values indicate shades of yellow.

**Figure 4:**
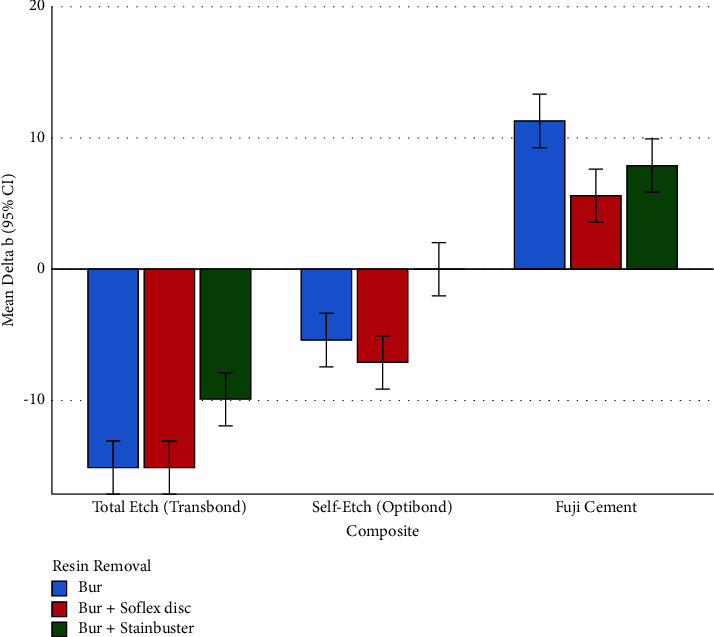
Means (and 95% CIs) for the Δb parameter in all the 9 groups (*n* = 9 × 10). Negative values indicate shades of blue, while positive values indicate shades of yellow.

**Figure 5:**
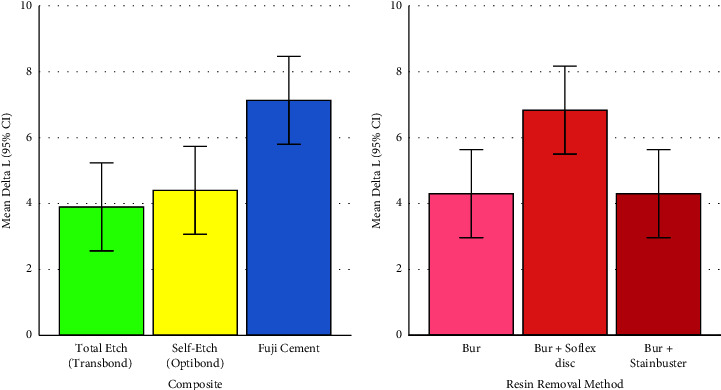
Means (and 95% CIs) for the ΔL parameter in 3 composites (*n* = 3 × 30) and 3 adhesive removal methods (*n* = 3 × 30). The values 0–100 indicate absolutely dark (0) to absolutely bright (100).

**Figure 6:**
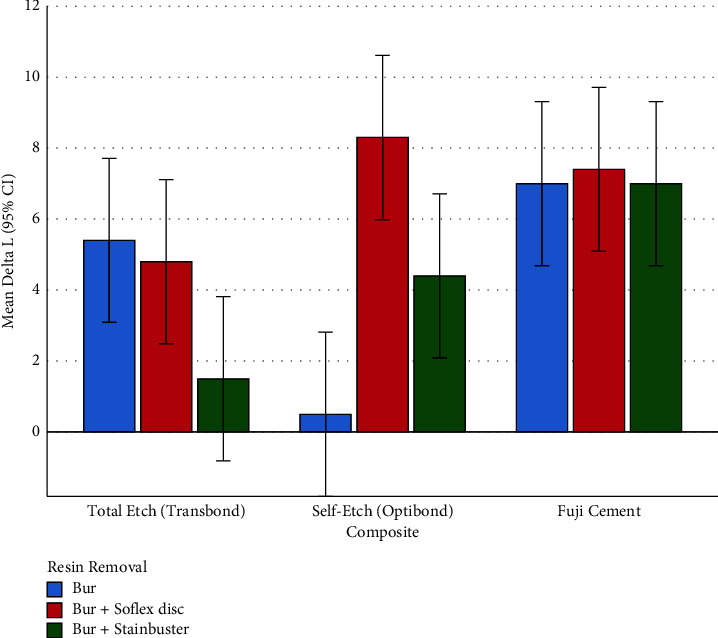
Means (and 95% CIs) for the ΔL parameter in all the 9 groups (*n* = 9 × 10). The values 0–100 indicate absolutely dark to absolutely bright, respectively.

**Figure 7:**
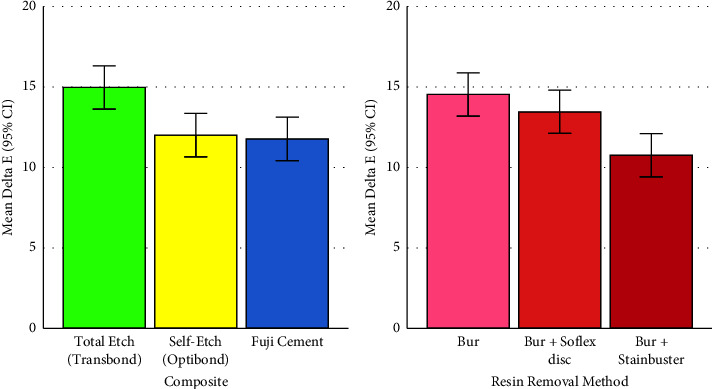
Means (and 95% CIs) for the ΔE parameter in 3 composites (*n* = 3 × 30) and 3 adhesive removal methods (*n* = 3 × 30).

**Figure 8:**
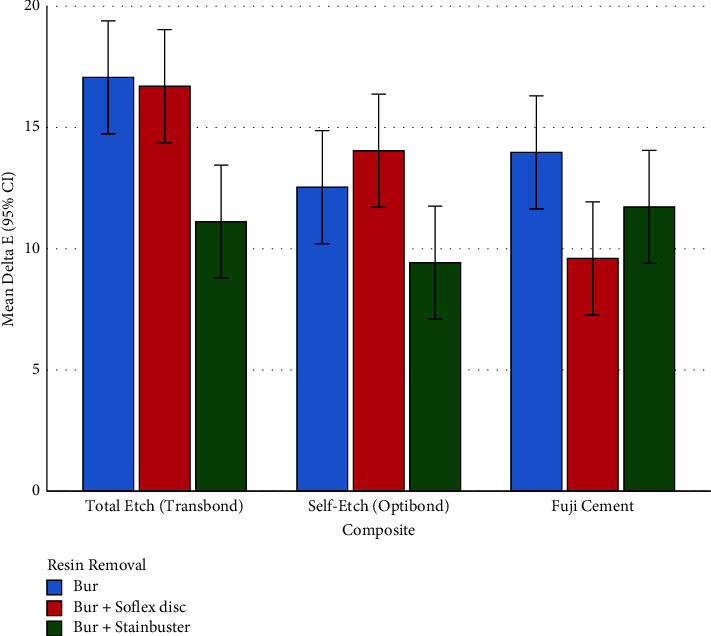
Means (and 95% CIs) for the ΔE parameter in all the 9 groups (*n* = 9 × 10).

There was no missing data. Descriptive statistics and 95% CIs are presented for various subgroups (*n* = 10), groups (*n* = 30), and the sample (*n* = 90) in Tables 1–3 and Figures 1–8. Subgroup analyses are demonstrated in Tables 3 and 4. The pairwise comparisons of 9 pairs of 3 adhesives against 3 resin removal methods are shown in Table 5. The one-sample ANOVA showed no significant comparisons between the baseline values of the subgroups (Table 3).

### 3.1. Delta a (Changes in the Green-Red Spectrum)

The two-way ANOVA showed a significant overall difference among the effects of different composites on the Δa parameter (*P* < 0.00000005, Figures 1 and 2, Tables 1 and 3). There was also a significant overall difference across the effects of adhesive removal methods on the Δa parameter (*P*=0.0000031, Figures 1 and 2, Tables 2 and 3). The interaction of composites and resin removal methods was as well significant, indicating that in different composites, the patterns of effects of resin removals may differ (*P*=0.0000031, Figure 2, Table 3).

#### 3.1.1. Pairwise Comparisons between Composites

In terms of composites' effects of the Δa parameter, the Tukey post hoc test showed significant pairwise comparisons between RMGI (Fuji) with each of the other composites (both *P* values ≤0.000003). However, the difference between total etch (Transbond) and self-etch (OptiBond) was insignificant (*P*=0.984, Figures 1 and 2, Table 1).

#### 3.1.2. Pairwise Comparisons between Adhesive Removal Methods

The Tukey post hoc test showed significant pairwise comparisons between the Δa parameter of group C (Bur + Stainbuster) with that of each of the other methods (both *P* values ≤0.0002). However, the difference between the Δa parameter of groups A (Bur) and B (Bur + Sof-Lex disc) was nonsignificant (*P*=0.696, Figures 1 and 2, Table 2).

#### 3.1.3. Subgroup Analyses for Δ*a* (Changes in the Green-Red Spectrum)

According to the one-way ANOVA, the parameter Δa was different among the 3 resin removal methods, only within the self-etch composite group (OptiBond, Table 3). The Tukey test showed that within the self-etch composite (OptiBond), the method of Bur + Stainbuster had Δa significantly different from the other resin removal methods (Table 4). However, the other two methods did not differ significantly from each other in terms of Δa (Table 4, Figure 2).

### 3.2. Delta b (Changes in the Blue-Yellow Spectrum)

According to the two-way ANOVA, the overall difference across the effects of different composites on the Δb parameter was significant (*P* < 0.00000005, Figures 3 and 4, Tables 1 and 3). Similarly, the overall difference among the effects of resin removal methods on the Δb parameter was significant (*P*=0.0000007, Figures 3 and 4, Tables 2 and 3). The interaction of these two variables was significant as well (*P*=0.0001710, Figure 4, Table 3).

#### 3.2.1. Pairwise Comparisons between Composites

All the 3 Tukey's pairwise comparisons between the Δb parameters of 3 composites became highly significant (all *P* values <0.00000005, Figures 3 and 4, Table 1).

#### 3.2.2. Pairwise Comparisons between Resin Removal Methods

All the 3 Tukey's pairwise comparisons between the Δb parameters of 3 composites became significant (all *P* values ≤0.014, Figures 3 and 4, Table 2).

#### 3.2.3. Subgroup Analyses for Δb (Changes in the Blue-Yellow Spectrum)

The parameter Δb was different among the 3 resin removal methods, within each of the 3 composites (Table 3). According to the Tukey test, within the total etch (Transbond) composite group, Bur + Stainbuster had a Δb significantly different from the other resin removal methods (Table 4). However, the Δb of the other two methods did not differ significantly from each other (Table 4). Similarly, within the self-etch composite group (OptiBond), Bur + Stainbuster had a Δb significantly different from the other adhesive removal methods (Table 4). But the other two methods did not differ significantly from each other in terms of Δb (Table 4). Within the RMGI (Fuji) composite group, the only significant pairwise comparison was between the Δb values of the Bur method versus the Bur + Sof-Lex disc (Table 4, Figure 4).

### 3.3. Delta L (Changes in Brightness)

The overall difference among the effects of different composites on the ΔL parameter was significant according to the two-way ANOVA (*P*=0.002, Figures 5 and 6, Tables 1 and 3). A significant overall difference was as well observed across the influences of adhesive removal methods on the ΔL parameter (*P*=0.011, Figures 5 and 6, Tables 2 and 3). Additionally, the interaction of composites and resin removal methods was significant (*P*=0.001, Figure 6, Table 3).

#### 3.3.1. Pairwise Comparisons between Composites

There were significant pairwise comparisons between the ΔL parameters of RMGI (Fuji) with each of the other composites (both Tukey's *P* values ≤0.014). Nevertheless, the difference between total etch (Transbond) and self-etch (OptiBond) was not significant (*P*=0.858, Figures 5 and 6, Table 1).

#### 3.3.2. Pairwise Comparisons between Resin Removal Methods

The pairwise comparisons between the ΔL parameter of group B (Bur + Sof-Lex disc) with the ΔL parameter each of the other methods were significant (both *P* values = 0.025). But the difference between groups A (Bur) and C (Bur + Stainbuster) was insignificant (*P*=0.696, Figures 5 and 6, Table 2).

#### 3.3.3. Subgroup Analyses for ΔL (Brightness Alterations)

The one-way ANOVA showed that ΔL was different among the 3 resin removal methods, only within the self-etch composite group (OptiBond, Table 3). The Tukey test showed that within the self-etch composite group (OptiBond), the only significant pairwise comparison was between the ΔL values of the Bur method versus the Bur + Sof-Lex disc (Table 4, Figure 6).

### 3.4. Delta E (the Overall Color Change)

The effects of different composites on the ΔE parameter were significantly different (two-way ANOVAs *P*=0.002, Figures 7 and 8, Tables 1 and 3). So were the effects of adhesive removal methods on the ΔE parameter (two-way ANOVAs *P*=0.001, Figures 7 and 8, Tables 2 and 3) as well as the interaction of composites and adhesive removal methods (*P*=0.008, Figure 8, Table 3).

#### 3.4.1. Pairwise Comparisons between Composites

There were significant pairwise comparisons between total etch (Transbond) and each of the other composites (both Tukey's *P* values ≤0.008). Nonetheless, the difference between self-etch (OptiBond) and RMGI (Fuji) was not significant (*P*=0.967, Figures 7 and 8, Table 1).

#### 3.4.2. Pairwise Comparisons between Adhesive Removal Methods

The Tukey test indicated significant pairwise comparisons between the ΔE parameter of group C (Bur + Stainbuster) with ΔE of each of the other methods (both *P* values ≤0.017). But the difference between ΔE of groups A (Bur) and B (Bur + Sof-Lex disc) was insignificant (*P*=0.500, Figures 7 and 8, Table 2).

#### 3.4.3. Subgroup Analyses for ΔE (Overall Discoloration)

The parameter ΔE was different among the 3 resin removal methods, within 2 of the composites (Table 3). Within the total etch (Transbond) composite group, Bur + Stainbuster had a ΔE significantly different from the other resin removal methods (Table 4). But the other two methods did not differ significantly from each other (Table 4). Within the RMGI (Fuji) composite group, the only significant pairwise comparison was between the ΔE values of the Bur method versus the Bur + Sof-Lex disc (Table 4, Figure 8).

#### 3.4.4. Comparing ΔE Values of 9 Subgroups (Pairs of Adhesives and Resin Removal Methods) with Each Other

The one-way ANOVA showed an overall significant difference across all the 9 groups (*P*=0.00001). The Tukey test detected 7 pairwise comparisons (Table 5, Figure 8). The smallest ΔE values were seen when self-etch (OptiBond) or total etch (Transbond) composites were removed using burs together with Stainbuster burs and also when RMGI (Fuji) was removed using Burs + Sof-Lex discs. The greatest ΔE values were observed when total etch (Transbond) was removed using burs with or without Sof-Lex discs.

#### 3.4.5. Comparing ΔE with the Values 1.0 and 3.7

The one-sample *t*-test showed that all the 9 mean ΔE values pertaining to the 9 groups were significantly greater than both 3.7 (all the 9 *P* values ≤0.002) and 1.0 (all the 9 *P* values <0.0005).

## 4. Discussion

The findings of the current study showed that all 9 groups caused clinically detectable color changes. This was similar to the first study on the possible changes in enamel color related to orthodontics [12], which showed clinically detectable color alterations (Δ*E* = 3.7) in all groups [12]. However, their choice of the adhesive system appeared to have insignificant effects on color changes [12]. The latter result was in contrast to our findings, showing that the adhesive in use can affect discoloration extent. The results of the present study showed that the use of different types of adhesives depending on their preparation method can have different effects on the colorability of tooth enamel. The use of RMGI resulted in the lowest amount of colorability, while the use of the 5th-generation total etch adhesive resulted in the highest amount of colorability. The observed difference may be attributed to the materials in use and even their brands, the rest of the methodological procedures (e.g., the staining methods, materials, and durations), and even the statistical limitations associated with smaller samples. Another study conducted by Joo et al. [21] on total etch and self-etch adhesives showed that the color change in the conventional acid etching adhesive was greater compared to the SEP adhesive (self-etching primers). A similar result was obtained in the present study. Another study showed no significant color change when using self-etch adhesives versus Fuji cement [12]. This was in agreement with this research as well. In the present study, the lowest amount of colorability was observed in these two adhesive groups, and no significant difference was observed between the two. In the study of Bucar et al. [22], it was shown that color change would be less when using the Fuji cement adhesive compared to the other ones [22], and the same result was obtained in the current study.

In a study by Boncuk et al. [23], 2 types of cleaning methods were used to remove adhesive residues in combination with 3 composites. The greatest color change occurred when only tungsten carbide burs were used. On the other hand, the lowest amount of color alteration was observed when the combination of a tungsten carbide bur and a Stainbuster bur was used [23]. Their results were consistent with the findings of this research. Also, their study [23] showed that the use of a Stainbuster bur to remove adhesives had the least effect on enamel discoloration, which resembled the results of the present study.

It was noteworthy that all interactions between all adhesives and all resin removal methods observed in this study were significant. This means that the results regarding the effects of adhesives on discoloration extents cannot be used without knowing the adhesive removal method, and similarly, the results pertaining to the adhesive removal methods would not be generalizable without specifying the used adhesive. This necessitates the assessment of each pair of adhesive plus resin removal methods separately because the overall coloration results may differ for each adhesive depending on the resin removal method to be followed. Hence, adhesive composition along with adhesive removal methods should be investigated in future evaluation for the effect on tooth enamel discoloration. It has been observed earlier that different techniques of adhesive removal together with the use of different generations and types of adhesive can have different effects on enamel discoloration [24]. The results of the current study showed that the use of the 5th generation adhesive with the total etch preparation method and tungsten carbide bur removal method had the highest amount of color change. Even though the use of 7th generation self-etch adhesive and Fuji cement together with the method of removing resin using tungsten carbide burs and then polishing with Stainbuster burs had the least amount of discoloration on tooth enamel. Ye et al. [24] asserted that the use of the glass-ionomer cement adhesive followed by resin removal using carbide burs and Sof-Lex polishers had the lowest colorability [24]. Very few studies are available in this regard, necessitating more examinations in the future.

On evaluating each colorimetric dimension independently, numerous notable results were obtained. In terms of Δa, it was found that, unlike RMGI which mildly tends to have redder shades, total etch and self-etch composites tend to have greener shades. Using burs with or without Sof-Lex discs would cause greener shades, while adding Stainbuster burs to conventional burs would shift the color to milder redder shades. Regarding Δb, unlike RMGI, which tends to have strong yellow shades, total etch and self-etch composites tend to have bluer shades. All the assessed resin removal methods would cause shades of blue (with Bur + Sof-Lex causing the greatest shades of blue and Bur + Stainbuster causing the mildest shades of blue). Concerning ΔL, RMGI resulted in the brightest ΔL changes, while brightness alterations of total etch and self-etch composites were darker and similar to each other. Moreover, Bur + Sof-Lex resulted in the brightest L changes, while the brightness alterations of Bur and Bur + Stainbuster were darker and similar to each other. Besides, the significant interactions would add to the complexity. The authors could not find any other study reporting each dimension of colorimetry independently to compare the results of the present study.

This study was limited by some factors. The sample size was not calculated using mathematical formulas; still, it should be noted that our sample size was much greater than that of many other colorimetry studies that had used a small number of specimens (3 or more) per group. In any study on dental materials, the results of each brand of materials in use might not be generalized to the same types of materials from other brands. Moreover, the *in vitro* nature of this study necessitates future clinical studies for verification of its results in the ever-changing oral environment.

An important point to note is that choosing proper adhesives and resin removal methods depends not only on the discoloration extent caused by these methods but also on other factors including proper bond strengths or enamel damage upon resin removal [4, 5, 7–10]. For example, glass ionomer cements may present significantly lower shear bond strengths compared to Transbond XT [10]. Unlike the discoloration caused by orthodontic adhesives and resin removal methods, which is underresearched, factors affecting bracket bond strengths are much more explored and determined [3–10]. Still, future studies are warranted to comparatively investigate the adhesives and resin removal methods presented in this study also in terms of their shear bond strengths and adhesive remnant indexes in order to enable clinicians to decide better.

## 5. Conclusions

All 9 pairs of adhesives and resin removal techniques will cause quite visible discolorations. Still, self-etch composites or RMGI might be recommended over total etch composites. Moreover, using Stainbuster burs together with tungsten carbide burs is recommended to reduce discoloration. However, it should be taken into consideration that the coloration caused by each composite type can change drastically given the adhesive removal technique used afterward. Therefore, it is advisable to consider pairs of “composite types together with resin removal methods” instead of relying on the results of composites or resin removal methods alone. In this regard, the composites recommended are self-etch or total etch composites removed by tungsten carbide burs together with Stainbuster burs and also RMGI composites removed using tungsten carbide together with Sof-Lex discs.

## Figures and Tables

**Table 1:** Descriptive statistics and 95% CIs for the assessed parameters in each composite group (*n* = 30), each resin removal group (*n* = 30), and the whole sample (*n* = 90).

Parameters	Groups	*N*	Mean	SD	95% CI		Min	Max
Initial a	Total Etch (Transbond)	30	4.67	2.02	3.91	5.42	0.00	8.00
	Self-Etch (OptiBond)	30	19.97	3.94	18.49	21.44	13.00	28.00
	RMGI (Fuji)	30	2.23	1.38	1.72	2.75	0.00	5.00
	All	90	8.96	8.33	7.21	10.70	0.00	28.00

Initial b	Total Etch (Transbond)	30	17.07	3.29	15.84	18.30	12.00	24.00
	Self-Etch (OptiBond)	30	4.27	1.23	3.81	4.73	2.00	7.00
	RMGI (Fuji)	30	18.40	4.11	16.87	19.93	8.00	27.00
	All	90	13.24	7.11	11.76	14.73	2.00	27.00

Initial L	Total Etch (Transbond)	30	78.87	4.10	77.34	80.40	70.00	85.00
	Self-Etch (OptiBond)	30	79.93	4.64	78.20	81.67	70.00	86.00
	RMGI (Fuji)	30	77.43	3.05	76.30	78.57	71.00	87.00
	All	90	78.74	4.07	77.89	79.60	70.00	87.00

Initial a	Bur	30	9.67	9.17	6.24	13.09	0.00	28.00
	Bur + Sof-Lex disc	30	9.17	8.24	6.09	12.24	0.00	25.00
	Bur + Stainbuster	30	8.03	7.70	5.16	10.91	0.00	26.00

Initial b	Bur	30	13.33	6.79	10.80	15.87	2.00	24.00
	Bur + Sof-Lex disc	30	13.43	7.15	10.76	16.10	3.00	24.00
	Bur + Stainbuster	30	12.97	7.61	10.13	15.81	3.00	27.00

Initial L	Bur	30	79.03	4.72	77.27	80.80	70.00	86.00
	Bur + Sof-Lex disc	30	78.63	3.29	77.41	79.86	75.00	86.00
	Bur + Stainbuster	30	78.57	4.20	77.00	80.13	70.00	87.00

SD, standard deviation; CI, confidence interval; Min, minimum; Max, maximum; RMGI, resin-modified glass ionomer.

**Table 2:** Descriptive statistics and 95% CIs for the assessed delta parameters in each composite group (*n* = 30), each resin removal group (*n* = 30), and the whole sample (*n* = 90).

Parameters	Groups	*N*	Mean	SD	95% CI		Min	Max
Δa	Total Etch (Transbond)	30	−3.43	2.22	−4.26	−2.60	−7.00	1.00
	Self-Etch (OptiBond)	30	−4.23	8.55	−7.43	−1.04	−19.00	11.00
	RMGI (Fuji)	30	1.77	1.28	1.29	2.24	−1.00	4.00
	All	90	−1.97	5.76	−3.17	−0.76	−19.00	11.00

Δb	Total Etch (Transbond)	30	−13.37	4.29	−14.97	−11.77	−20.00	−5.00
	Self-Etch (OptiBond)	30	−4.17	3.79	−5.58	−2.75	−9.00	3.00
	RMGI (Fuji)	30	8.27	4.21	6.69	9.84	2.00	21.00
	All	90	−3.09	9.79	−5.14	−1.04	−20.00	21.00

ΔL	Total Etch (Transbond)	30	3.90	4.09	2.37	5.43	−4.00	16.00
	Self-Etch (OptiBond)	30	4.40	5.22	2.45	6.35	−3.00	19.00
	RMGI (Fuji)	30	7.13	2.70	6.13	8.14	2.00	12.00
	All	90	5.14	4.33	4.24	6.05	−4.00	19.00

ΔE	Total Etch (Transbond)	30	14.96	4.60	13.25	16.68	6.16	22.14
	Self-Etch (OptiBond)	30	12.01	4.71	10.25	13.77	4.12	21.12
	RMGI (Fuji)	30	11.77	3.14	10.60	12.94	6.16	23.19
	All	90	12.91	4.41	11.99	13.84	4.12	23.19

Δa	Bur	30	−3.90	6.71	−6.40	−1.40	−19.00	4.00
	Bur + Sof-Lex disc	30	−3.10	4.25	−4.69	−1.51	−14.00	2.00
	Bur + Stainbuster	30	1.10	4.87	−0.72	2.92	−7.00	11.00

Δb	Bur	30	−3.07	11.54	−7.38	1.24	−19.00	21.00
	Bur + Sof-Lex disc	30	−5.53	9.00	−8.89	−2.17	−20.00	10.00
	Bur + Stainbuster	30	−0.67	8.24	−3.74	2.41	−17.00	14.00

ΔL	Bur	30	4.30	4.32	2.69	5.91	−3.00	16.00
	Bur + Sof-Lex disc	30	6.83	4.02	5.33	8.33	−1.00	19.00
	Bur + Stainbuster	30	4.30	4.28	2.70	5.90	−4.00	12.00

ΔE	Bur	30	14.53	4.80	12.74	16.33	4.12	23.19
	Bur + Sof-Lex disc	30	13.45	4.22	11.88	15.03	7.14	22.14
	Bur + Stainbuster	30	10.76	3.32	9.52	12.00	5.48	18.87

SD, standard deviation; CI, confidence interval; Min, minimum; Max, maximum; RMGI, resin-modified glass ionomer.

**Table 3:** Descriptive statistics and 95% CIs for the assessed parameters in each subgroup (*n* = 10), as well as the results of subgroup analyses using the one-way ANOVA for the delta values.

Composites	Parameters	Resin removal	*N*	Mean	SD	95% CI		Min	Max	*P*
Total Etch (Transbond)	Initial a	Bur	10	4.70	1.77	3.44	5.96	2.00	7.00	0.260
		Bur + Sof-Lex disc	10	5.40	1.96	4.00	6.80	2.00	8.00	
		Bur + Stainbuster	10	3.90	2.23	2.30	5.50	0.00	7.00	
	Initial b	Bur	10	16.70	2.26	15.08	18.32	13.00	19.00	0.220
		Bur + Sof-Lex disc	10	18.50	4.50	15.28	21.72	12.00	24.00	
		Bur + Stainbuster	10	16.00	2.40	14.28	17.72	13.00	19.00	
	Initial L	Bur	10	79.60	4.38	76.47	82.73	70.00	85.00	0.502
		Bur + Sof-Lex disc	10	79.40	2.84	77.37	81.43	76.00	83.00	
		Bur + Stainbuster	10	77.60	4.93	74.08	81.12	70.00	85.00	
	Δa	Bur	10	−3.40	1.96	−4.80	−2.00	−7.00	0.00	0.678
		Bur + Sof-Lex disc	10	−3.90	2.18	−5.46	−2.34	−6.00	0.00	
		Bur + Stainbuster	10	−3.00	2.62	−4.88	−1.12	−6.00	1.00	
	Δb	Bur	10	−15.10	3.11	−17.32	−12.88	−19.00	−11.00	**0.004**
		Bur + Sof-Lex disc	10	−15.10	3.35	−17.50	−12.70	−20.00	−8.00	
		Bur + Stainbuster	10	−9.90	4.28	−12.96	−6.84	−17.00	−5.00	
	ΔL	Bur	10	5.40	4.79	1.97	8.83	1.00	16.00	0.066
		Bur + Sof-Lex disc	10	4.80	2.82	2.78	6.82	−1.00	9.00	
		Bur + Stainbuster	10	1.50	3.63	−1.10	4.10	−4.00	8.00	
	ΔE	Bur	10	17.07	3.36	14.66	19.47	11.45	20.27	**0.002**
		Bur + Sof-Lex disc	10	16.71	3.10	14.49	18.93	10.44	22.14	
		Bur + Stainbuster	10	11.12	4.74	7.73	14.51	6.16	18.87	

Self-Etch (OptiBond)	Initial a	Bur	10	21.70	4.64	18.38	25.02	14.00	28.00	0.122
		Bur + Sof-Lex disc	10	20.10	2.73	18.15	22.05	17.00	25.00	
		Bur + Stainbuster	10	18.10	3.73	15.44	20.76	13.00	26.00	
	Initial b	Bur	10	4.70	1.64	3.53	5.87	2.00	7.00	0.269
		Bur + Sof-Lex disc	10	4.30	0.82	3.71	4.89	3.00	6.00	
		Bur + Stainbuster	10	3.80	1.03	3.06	4.54	3.00	6.00	
	Initial L	Bur	10	80.40	6.06	76.07	84.73	70.00	86.00	0.928
		Bur + Sof-Lex disc	10	79.80	4.21	76.79	82.81	75.00	86.00	
		Bur + Stainbuster	10	79.60	3.81	76.88	82.32	75.00	85.00	
	Δa	Bur	10	−10.40	7.31	−15.63	−5.17	−19.00	0.00	**0.000039**
		Bur + Sof-Lex disc	10	−6.40	4.55	−9.66	−3.14	−14.00	1.00	
		Bur + Stainbuster	10	4.10	6.08	−0.25	8.45	−7.00	11.00	
	Δb	Bur	10	−5.40	1.71	−6.63	−4.17	−8.00	−2.00	**<0.0000005**
		Bur + Sof-Lex disc	10	−7.10	1.29	−8.02	−6.18	−9.00	−5.00	
		Bur + Stainbuster	10	0.00	3.33	−2.38	2.38	−6.00	3.00	
	ΔL	Bur	10	0.50	2.17	−1.05	2.05	−3.00	5.00	**0.001**
		Bur + Sof-Lex disc	10	8.30	5.89	4.09	12.51	−1.00	19.00	
		Bur + Stainbuster	10	4.40	3.84	1.66	7.14	−2.00	8.00	
	ΔE	Bur	10	12.55	6.24	8.08	17.01	4.12	20.71	0.077
		Bur + Sof-Lex disc	10	14.05	3.99	11.19	16.90	7.14	21.12	
		Bur + Stainbuster	10	9.43	2.05	7.96	10.90	5.48	11.36	

RMGI (Fuji)	Initial a	Bur	10	2.60	1.58	1.47	3.73	0.00	5.00	0.598
		Bur + Sof-Lex disc	10	2.00	1.41	0.99	3.01	0.00	4.00	
		Bur + Stainbuster	10	2.10	1.20	1.24	2.96	0.00	4.00	
	Initial b	Bur	10	18.60	3.81	15.88	21.32	13.00	24.00	0.687
		Bur + Sof-Lex disc	10	17.50	2.01	16.06	18.94	15.00	20.00	
		Bur + Stainbuster	10	19.10	5.86	14.91	23.29	8.00	27.00	
	Initial L	Bur	10	77.10	3.07	74.90	79.30	71.00	81.00	0.396
		Bur + Sof-Lex disc	10	76.70	1.64	75.53	77.87	75.00	79.00	
		Bur + Stainbuster	10	78.50	3.98	75.65	81.35	73.00	87.00	
	Δa	Bur	10	2.10	0.74	1.57	2.63	1.00	4.00	0.061
		Bur + Sof-Lex disc	10	1.00	1.05	0.25	1.75	−1.00	2.00	
		Bur + Stainbuster	10	2.20	1.62	1.04	3.36	0.00	4.00	
	Δb	Bur	10	11.30	4.52	8.06	14.54	5.00	21.00	**0.005**
		Bur + Sof-Lex disc	10	5.60	2.41	3.87	7.33	2.00	10.00	
		Bur + Stainbuster	10	7.90	3.54	5.37	10.43	5.00	14.00	
	ΔL	Bur	10	7.00	2.67	5.09	8.91	3.00	11.00	0.934
		Bur + Sof-Lex disc	10	7.40	1.43	6.38	8.42	5.00	9.00	
		Bur + Stainbuster	10	7.00	3.77	4.30	9.70	2.00	12.00	
	ΔE	Bur	10	13.98	3.49	11.48	16.48	10.68	23.19	**0.004**
		Bur + Sof-Lex disc	10	9.61	1.80	8.32	10.90	7.87	12.96	
		Bur + Stainbuster	10	11.73	2.37	10.04	13.43	6.16	14.70	

SD, standard deviation; CI, confidence interval; Min, minimum; Max, maximum; RMGI, resin-modified glass ionomer. Significant *P* values are given in bold font.

**Table 4:** Pairwise comparisons performed using the Tukey test following significant ANOVAs applied for subgroup analyses (reported in Table 3).

Composite	Parameter	Resin removal *I*	Resin removal *J*	Diff (*I − J*)	*P*	95% CI	
Total Etch (Transbond)	Δb	Bur	Bur + Sof-Lex disc	0.00	1.000	−4.01	4.01
			Bur + Stainbuster	−5.20	**0.009**	−9.21	−1.19
		Bur + Sof-Lex disc	Bur + Stainbuster	−5.20	**0.009**	−9.21	−1.19
	ΔE	Bur	Bur + Sof-Lex disc	0.36	0.975	−3.85	4.58
			Bur + Stainbuster	5.95	**0.005**	1.74	10.17
		Bur + Sof-Lex disc	Bur + Stainbuster	5.59	**0.008**	1.37	9.80

Self-Etch (OptiBond)	Δa	Bur	Bur + Sof-Lex disc	−4.00	0.321	−10.75	2.75
			Bur + Stainbuster	−14.50	**<0.0005**	−21.25	−7.75
		Bur + Sof-Lex disc	Bur + Stainbuster	−10.50	**0.002**	−17.25	−3.75
	Δb	Bur	Bur + Sof-Lex disc	1.70	0.238	−0.84	4.24
			Bur + Stainbuster	−5.40	**<0.0005**	−7.94	−2.86
		Bur + Sof-Lex disc	Bur + Stainbuster	−7.10	**<0.0005**	−9.64	−4.56
	ΔL	Bur	Bur + Sof-Lex disc	−7.80	**0.001**	−12.51	−3.09
			Bur + Stainbuster	−3.90	0.119	−8.61	0.81
		Bur + Sof-Lex disc	Bur + Stainbuster	3.90	0.119	−0.81	8.61

RMGI (Fuji)	Δb	Bur	Bur + Sof-Lex disc	5.70	**0.004**	1.71	9.69
			Bur + Stainbuster	3.40	0.106	−0.59	7.39
		Bur + Sof-Lex disc	Bur + Stainbuster	−2.30	0.340	−6.29	1.69
	ΔE	Bur	Bur + Sof-Lex disc	4.37	**0.003**	1.43	7.31
			Bur + Stainbuster	2.25	0.159	−0.69	5.18
		Bur + Sof-Lex disc	Bur + Stainbuster	−2.13	0.190	−5.06	0.81

Diff, difference; CI, confidence interval. Significant *P* values are given in bold font.

**Table 5:** The results of the Tukey post hoc test, comparing the 9 groups in terms of their overall changes in colorimetry indices (ΔE).

Pair *I*	Pair *J*	Diff (*I − J*)	*P*	95% CI	
Self-etch (OptiBond) removed by bur	Total etch (Transbond) removed by bur	−4.52	0.154	−9.80	0.76
RMGI (Fuji) removed by bur	Total etch (Transbond) removed by bur	−3.09	0.639	−8.37	2.19
	Self-etch (OptiBond) removed by bur	1.43	0.994	−3.85	6.71

Total etch (Transbond) removed by bur + Sof-Lex	Total etch (Transbond) removed by bur	−0.36	1.0	−5.64	4.92
	Self-etch (OptiBond) removed by bur	4.16	0.242	−1.12	9.44
	RMGI (Fuji) removed by bur	2.73	0.776	−2.55	8.01

Self-etch (OptiBond) removed by bur + Sof-Lex	Total etch (transbond) removed by bur	−3.02	0.666	−8.30	2.26
	Self-etch (OptiBond) removed by bur	1.50	0.992	−3.78	6.78
	RMGI (Fuji) removed by bur	0.07	1.0	−5.21	5.35
	Total etch (Transbond) removed by bur + Sof-Lex	−2.66	0.798	−7.94	2.62

RMGI (Fuji) removed by bur + Sof-Lex	Total etch (Transbond) removed by bur	−7.46	**0.001**	−12.74	−2.18
	Self-etch (OptiBond) removed by bur	−2.94	0.698	−8.22	2.34
	RMGI (Fuji) removed by bur	−4.37	0.187	−9.65	0.91
	Total etch (Transbond) removed by bur + Sof-Lex	−7.10	**0.002**	−12.38	−1.82
	Self-etch (OptiBond) removed by bur + Sof-Lex	−4.44	0.172	−9.72	0.84

Total etch (Transbond) removed by bur + Stainbuster	Total etch (Transbond) removed by bur	−5.95	**0.016**	−11.23	−0.67
	Self-etch (OptiBond) removed by bur	−1.43	0.994	−6.71	3.85
	RMGI (Fuji) removed by bur	−2.86	0.728	−8.14	2.42
	Total etch (Transbond) removed by bur + Sof-Lex	−5.59	**0.030**	−10.87	−0.31
	Self-etch (OptiBond) removed by bur + Sof-Lex	−2.93	0.703	−8.21	2.35
	RMGI (Fuji) removed by bur + Sof-Lex	1.51	0.992	−3.77	6.79

Self-etch (OptiBond) removed by bur + Stainbuster	Total etch (Transbond) removed by bur	−7.64	**<0.0005**	−12.92	−2.36
	Self-etch (OptiBond) removed by bur	−3.12	0.628	−8.40	2.16
	RMGI (Fuji) removed by bur	−4.55	0.148	−9.83	0.73
	Total etch (Transbond) removed by bur + Sof-Lex	−7.28	**0.001**	−12.56	−2.00
	Self-etch (OptiBond) removed by bur + Sof-Lex	−4.62	0.135	−9.90	0.66
	RMGI (Fuji) removed by bur + Sof-Lex	−0.18	1.0	−5.46	5.10
	Total etch (Transbond) removed by bur + Stainbuster	−1.69	0.983	−6.97	3.59

RMGI (Fuji) removed by bur + Stainbuster	Total etch (Transbond) removed by bur	−5.34	**0.046**	−10.62	−0.06
	Self-etch (OptiBond) removed by bur	−0.81	1.0	−6.09	4.47
	RMGI (Fuji) removed by bur	−2.25	0.911	−7.53	3.03
	Total etch (Transbond) removed by bur + Sof-Lex	−4.97	0.081	−10.25	0.31
	Self-etch (OptiBond) removed by bur + Sof-Lex	−2.31	0.896	−7.59	2.97
	RMGI (Fuji) removed by bur + Sof-Lex	2.13	0.933	−3.15	7.41
	Total etch (Transbond) removed by bur + Stainbuster	0.61	1.0	−4.67	5.89
	Self-etch (OptiBond) removed by bur + Stainbuster	2.30	0.898	−2.98	7.58

Diff, difference; CI, confidence interval; RMGI, resin-modified glass ionomer. Significant *P* values are given in bold font.

## Data Availability

The data are available from the corresponding author upon request.
